# Improving Recognition Accuracy of Pesticides in Groundwater by Applying TrAdaBoost Transfer Learning Method

**DOI:** 10.3390/s23083856

**Published:** 2023-04-10

**Authors:** Donghui Chen, Bingyang Wang, Xiao Yang, Xiaohui Weng, Zhiyong Chang

**Affiliations:** 1 Key Laboratory of Bionic Engineering, Ministry of Education, Jilin University, Changchun 130022, China; 2 College of Biological and Agricultural Engineering, Jilin University, Changchun 130022, China; 3 Weihai Institute for Bionics, Jilin University, Weihai 264401, China; 4 School of Mechanical and Aerospace Engineering, Jilin University, Changchun 130022, China

**Keywords:** electronic nose, groundwater, pesticide, support vector machine, TrAdaBoost

## Abstract

Accurate and rapid prediction of pesticides in groundwater is important to protect human health. Thus, an electronic nose was used to recognize pesticides in groundwater. However, the e-nose response signals for pesticides are different in groundwater samples from various regions, so a prediction model built on one region’s samples might be ineffective when tested in another. Moreover, the establishment of a new prediction model requires a large number of sample data, which will cost too much resources and time. To resolve this issue, this study introduced the TrAdaBoost transfer learning method to recognize the pesticide in groundwater using the e-nose. The main work was divided into two steps: (1) qualitatively checking the pesticide type and (2) semi-quantitatively predicting the pesticide concentration. The support vector machine integrated with the TrAdaBoost was adopted to complete these two steps, and the recognition rate can be 19.3% and 22.2% higher than that of methods without transfer learning. These results demonstrated the potential of the TrAdaBoost based on support vector machine approaches in recognizing the pesticide in groundwater when there were few samples in the target domain.

## 1. Introduction

Groundwater is an important source of fresh water in the world. More than 1.5 billion people worldwide depend on groundwater as their drinking water source [1]. However, the use of pesticides, fertilizers, and other pollutants led to groundwater pollution, among which pesticide was the most harmful pollutant for aquatic environments [2]. The presence of pesticides in groundwater may cause great harm to the human body, through the food chain, such as mutagenic, cancer, and infertility [3,4]. According to FAO (FAO, 2018), more than 26 million people worldwide are poisoned by pesticides every year, causing losses of about 8 billion dollars annually. In this sense, the early detection of pesticides in groundwater can effectively prevent these pesticides from entering the food chain, and rapid remedial actions can be taken.

Traditionally, pesticides in groundwater can be qualitatively and quantitatively detected by high-performance liquid chromatography (HPLC), gas chromatography–mass spectrometry (GC-MS), and liquid chromatography–mass spectrometry (LC-MS) [5,6,7]. The detection results of these methods have good sensitivity and stability. However, the shortcomings such as large-volume, time-consuming, complex extraction procedures, and analytical techniques restrict their on-site and real-time application [8]. Thus, many researchers are committed to developing simple and rapid pesticide detection instruments.

The electronic nose (E-nose) is a rapid detection instrument that has been rising in recent years [9,10]. The instrument is composed of a sensor array with several different sensors and some machine learning algorithms, which are capable of recognizing simple or complex odors [11,12]. Given their rapid recognition ability while still being compact and lightweight, this technique has been attracting extensive attention in the pesticide detection field [13]. Amoker et al. realized the prediction of pesticides on the surface of mint based on the developed three-sensor e-nose system [14]. Tang et al. used principal component analysis (PCA), linear discriminant analysis (LDA) together with a support vector machine (SVM) to evaluate the concentrations of cypermethrin and chlorpyrifos pesticides on apples by PEN3 e-nose [15]. Nategh et al. used a self-made e-nose system to realize the identification of whether four kinds of cherries with different maturity were polluted by pesticides [16]. Tang et al. [17] characterized and differentiated teas containing different concentrations of pyrethroid pesticides by e-nose using a back propagation neural network (BPNN). To sum up, the e-nose has successfully achieved pesticide detection in the food field. However, there are still some challenges in the detection of pesticide residues in groundwater by e-nose; thus, the report about using e-nose to detect pesticides in groundwater is rare.

At present, the application of e-nose in the groundwater pesticide detection field is mainly limited in two aspects: (1) The training model cannot be transferred, and (2) limited target samples. For the same pesticide, the e-nose response signals collected in two different regions are distributed differently, which is caused by the e-nose being subjected to other interfering volatiles (fertilizers, chemicals) in the groundwater [18]. Thus, the recognition accuracy of the model trained in region A is reduced when used to predict samples from region B. However, it is often impractical to gain enough groundwater samples at each region to develop new prediction models, especially when the cost of groundwater sample collection and calibration is high. How to use old samples to solve the problem of insufficient target samples is an urgent issue. The transfer learning (TL) method is mainly used to acquire knowledge from the old data domain to solve problems in the new data domain [19]. Thus, this paper proposed to test TL feasibility for building groundwater pesticide prediction models.

The instance-based TL method, TrAdaBoost [20], was employed in this study since they meet the requirements of groundwater pesticide prediction model building. The first challenge to building a groundwater pesticide prediction model is that the source data (old data) borrowed from other regions may not be applicable. TrAdaBoost, however, allows the distributions to be different between source and target domain data, as it can extract the valuable parts from the source data by adjusting the data weights. The second challenge is that limited target data are usable; it is not appropriate to collect and calibrate data by consuming time and resources. TrAdaBoost can employ a small quantity of target data and the useful parts of source domain data to build a powerful prediction model [21]. As TrAdaBoost is suitable for data sets with different distributions and small sample sizes, it makes sense to use the TrAdaBoost method to build groundwater pesticide prediction models.

Currently, the TrAdaBoost method has not been applied to the e-nose detection field, which is also an innovation of this study. In other fields, the TrAdaBoost algorithm has been widely used, such as safety accident prediction, mechanical device failure detection, disease diagnosis, and agricultural product monitoring. Fu et al. proposed using the TrAdaBoost method to improve the performance of the landslide susceptibility model with limited samples. The experimental results showed that the method can improve the prediction accuracy of the model by up to 10% [22]. Jin et al. applied the TrAdaBoost method to address the problem of poor prediction results of wind turbine operating conditions due to insufficient data [23]. When only a few data are available, the diagnosis of a disease might be difficult. Fu et al. suggested using data from the source domain to assist in disease diagnosis, which can be implemented by the TrAdaBoost method. The feasibility of this method has been verified [24]. Xun et al. combined a moderate-resolution imaging spectroradiometer with the TrAdaBoost algorithm to realize the prediction of cotton planting areas in different regions using a few samples in the target domain [25]. The above research results showed that the TrAdaBoost method could establish a good prediction model when the target domain samples were limited. These researches provide support for our work.

The purpose of this study is to address the problem of how to improve pesticide predicting accuracy in groundwater with limited target domain samples. The solution to this problem can not only promote the application of e-noses in detecting groundwater pesticides but also have a certain value for the entire e-nose industry. In this paper, the soil leaching experiment was used to simulate two different regions of groundwater samples (source domain and target domain), including samples polluted and unpolluted by pesticides. The odor information data of the pesticide in groundwater samples were gathered by an e-nose developed by our team. When the target domain samples are limited, the recognition accuracy of the model trained by the e-nose is low, and the source domain sample cannot be used to assist in training the prediction model due to the significant difference in the response signal between the source and target domain samples. Therefore, the TrAdaBoost algorithm was introduced to process source domain data so that it can be used to assist the target domain data in building a good prediction model for pesticides in groundwater. The main contributions of this study are as follows:

- The TrAdaBoost algorithm was first applied to the e-nose field.
- The method of PCA, multi-feature extraction algorithms combined with an SVM classifier, were applied to prove that the response signals of source and target domain samples have significant differences.
- Two parameters of the TrAdaBoost algorithm in the pesticide recognition process were optimized: the number of iterations and the number of source domain samples participating in model training.
- The e-nose system applied the TrAdaBoost algorithm to realize qualitative and semi-quantitative identification of pesticides in groundwater under the condition of limited target domain data.

The sections of this work are arranged as follows. In Section 2, we introduced the preparation of experimental samples, the composition of the e-nose system, and the data analysis methods used in this work, including the TrAdaBoost algorithm. Section 3 evaluated the performance of the TrAdaBoost algorithm based on source and target domain datasets collected by the e-nose system and conducted a qualitative and semi-quantitative analysis of pesticides in the target domain. Section 4 provided the conclusion and discussed the limitations and future directions of this research.

## 2. Materials and Methods

### 2.1. Sample Preparation

#### 2.1.1. Soil Sample

The soil samples used in this paper were from the farmland of Weihai, Shandong Province (37°20′ N latitude and 122°05′ E, location 1) and the experimental farmland of Jilin University in Changchun, Jilin Province (43°87′ N latitude and 125.33′ E, location 2). These two representative regions are important grain-producing areas in China, and they need to consume a large amount of pesticides every year. Before conducting the soil leaching experiment, the soil samples were dried, crushed, and sieved into 1 mm to ensure uniformity. The properties of the soil samples are shown in Table 1.

#### 2.1.2. Pesticide Reagent

Pesticides were obtained from the pesticide market in Changchun, Jilin Province, China, and Tanmo Quality Inspection Technology Co., Ltd. in Changzhou, Jiangsu Province, China. The pesticides were chlorpyrifos (40%), malathion (70%), chlorothalonil (75%), and lindane (99%), which were the detection indicators of groundwater quality (GB/T 14848-2017) [26]. Headspace gas chromatography–mass spectrometry (HS-GC/MS) was used to determine volatile compounds in the pesticides, and the results are shown in Table S1. The analysis of volatile compounds provided strong support for the application of e-nose to detect pesticide types.

#### 2.1.3. Groundwater Sample Preparation

**Figure 1:**
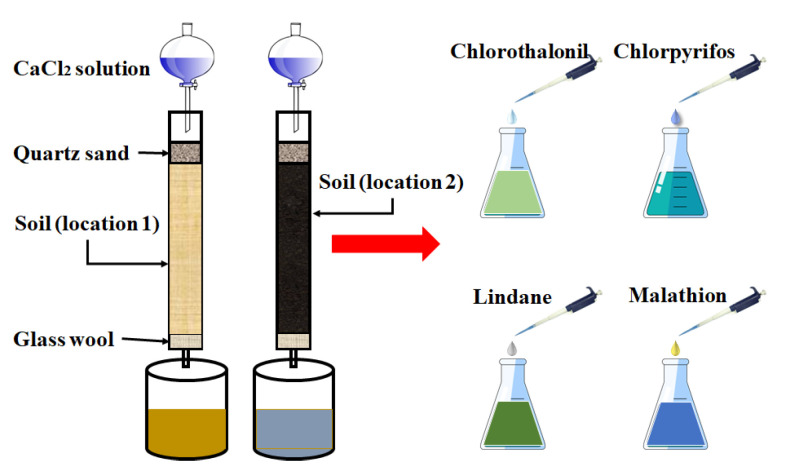
The diagram for preparation of groundwater samples containing pesticides.

Pollutants in groundwater are mainly transported from polluted soil via leaching or percolating processes [27]. Thus, groundwater samples were prepared by soil leaching experiment in this study. Predecessors used this method to simulate and study groundwater pollution [28,29]. The process of sample preparation is shown in Figure 1. Firstly, 0.57 kg of soil was filled into the leaching column and compacted to make the soil column 40 cm high, which was in line with the bulk density of the soil under natural conditions. Secondly, 800 mL CaCl_2_ solution (0.01 mol/L) was used to simulate rainfall. Finally, groundwater samples (leachate) polluted by different pesticides were prepared by adding pesticides to groundwater samples. The soil samples (locations 1 and 2) were used to perform soil leaching experiments to obtain two sets of leachate (simulated groundwater samples 1 and 2). The volatile compounds in simulated groundwater samples were also determined by HS-GC/MS (Table S2). The result in Table S2 showed that different volatile compounds would be generated in groundwater due to the different soil. This is the reason why the recognition accuracy of the e-nose decreases. For preparing experimental samples, pesticides (chlorpyrifos, malathion, chlorothalonil, and lindane) were added to groundwater samples 1 and 2 to prepare pesticide-polluted groundwater samples (target domain samples, source domain samples). Three different concentrations of polluted samples were prepared: 100 μg/L, 500 μg/L, and 1000 μg/L. A total of 20 mL sample solution was put into a 100 mL conical flask and sealed with film as one detection sample.

In this study, a total of 1040 groundwater samples were prepared, including 520 source domain samples and 520 target domain samples. Each domain consisted of 480 samples polluted by pesticides and 40 samples unpolluted by pesticides. The 480 pesticide-polluted samples could be divided into 4 classes based on pesticide type, with 120 samples per class. Each class of samples contained 3 different pesticide concentration samples, with 40 samples for each concentration.

### 2.2. E-Nose System and Process

**Figure 2:**
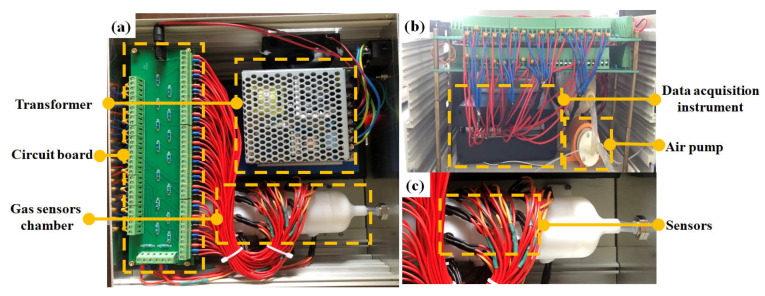
The structure of e-nose system. (**a**) Vertical view of e-nose, (**b**) left view of e-nose, (**c**) enlarged view of the gas sensors chamber.

The e-nose system was self-developed by our team. As shown in Figure 2, the e-nose was composed of a transformer, circuit board, gas sensors chamber, data acquisition instrument, air pump, and sensors. Because there were many volatile compounds in pesticides (Table S1), 26 MOS sensors (Table S3) were equipped with e-noses. To make the sensors work normally, it was necessary to configure a simple regulating circuit for the sensors, and the circuit also needed to be given an input voltage. In this study, the circuit was recommended by the manufacturer, and the input voltage of the circuit was 5 V voltage converted by the transformer. The effective power consumption and weight of the e-nose system are about 14.805 W and 2.5 kg.

**Figure 3:**
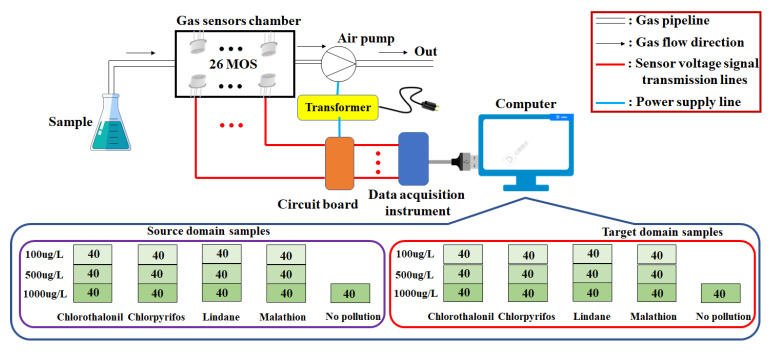
The flow chart of sample detection by e-nose. The transformer converted 220 V voltage into 5 V voltage to serve as an electric power supply device for the air pump and conditioning circuit on the circuit board. The data acquisition instrument was connected to the computer via USB to obtain electric power. The air pump served as a power source to transport the gas emitted from the sample to the gas sensors chamber. In the chamber, the gas would react with the sensor material at the surface, causing the sensor resistance to change, which in turn led to the output voltage of the conditioning circuit changing. The output voltages were read and converted into digital signals by the data acquisition instrument. The digital signals were stored in the computer. The data in the figure represented the type and number of the detection samples in this study.

The detection process was divided into three stages. Firstly, the sealed sample was stood for 15 min to make the headspace gas fill the conical flask. The change in temperature may cause a poor recognition accuracy of the e-nose. For this reason, the experiment was conducted at a room temperature of 18–22 °C to reduce the effect of temperature on the experimental results. Secondly, the intake pipe of the e-nose was inserted into the conical flask. The sampling time and frequency were set to 60 s and 100 HZ, respectively, and the flow rate of the air pump was 300 mL/min. A short sampling time might reduce the recognition accuracy of the e-nose system, while quite a long sampling time might not benefit from improving the recognition accuracy and would waste power. The sampling time of 60 s was sufficient to meet the needs of the experiment. Finally, before the next detection, clean air was used to clean the gas sensors chamber for 5 min to ensure that the e-nose response signal returned to the baseline location. The detection flow chart is shown in Figure 3. A total of 1040 sample data were collected at this stage. Each data were a matrix that included 6000 rows (60 s × 100 HZ) and 26 columns (number of sensors). Each statistic in the matrix represented the output voltage of the conditioning circuit at a certain time.

### 2.3. Data Analysis

Data analysis consisted primarily of two stages: feature extraction and pattern recognition.

#### 2.3.1. Feature Extraction

Feature extraction can reduce the data dimension, remove irrelevant and redundant data, and increase the effect of recognition. In this study, two types of feature extraction methods were applied: transient-state feature extraction and steady-state feature extraction method [30,31].

Transient state feature extraction methods:

- Fourier transform (FT): the response signals were used for Fourier transform, and the transform coefficients were applied as the feature values.
- Wavelet transform (WT): the response signals were used for wavelet transform, and the transform coefficients were applied as the feature values.
- Steady-state feature extraction methods:
- Integral value (IV): calculating the area below the sensor response curve.
- Max value (MAX): selecting the maximum voltage value from the e-nose response signal.
- Mean value (Mean): calculating the average voltage value for the e-nose response signal.

#### 2.3.2. Pattern Recognition

**Figure 4:**
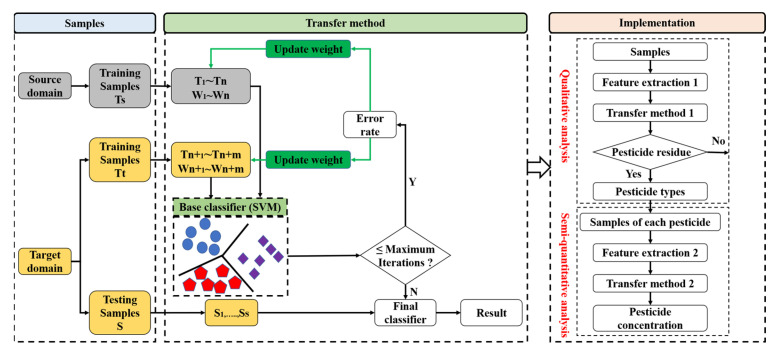
The flowchart of the methodology applied for identifying the pesticides in groundwater.

The flowchart of the methodology for identifying the pesticides in groundwater is shown in Figure 4. The first step was to use the TrAdaBoost transfer learning method to determine whether the groundwater was polluted by pesticides. If so, what was the type of pesticide? The second step was to apply this method to estimate the pesticide concentration in groundwater. The results of these two steps were evaluated by recognition accuracy.

SVM is a supervised classifier based on the kernel function. The basic idea of SVM is to convert the sample data from low-dimensional space to high-dimensional space, then establish an optimal hyperplane to maximize the distance between different classes of samples. Due to the special data processing process, the SVM is suitable for addressing nonlinear, small-sample, and high-dimensional sample problems [32]. Linear kernel function-based SVM was used in this paper.

TrAdaBoost is an effective transfer learning method. This method can filter out those data in the source domain, which are not helpful for sample recognition in the target domain. If data in the source domain are wrongly predicted in an iteration, their weight is decreased in the next iteration, and conversely, the data weight in the target domain will increase [20]. Due to the unique learning process, the TrAdaBoost algorithm has strong transmission ability and good convergence, and it is very suitable for processing data with similar distributions in the source and target domains [33]. These methods were performed by MATLAB 2020a (The Mathworks Inc., Natick, MA, USA).

## 3. Results and Discussion

### 3.1. PCA Analysis

**Figure 5:**
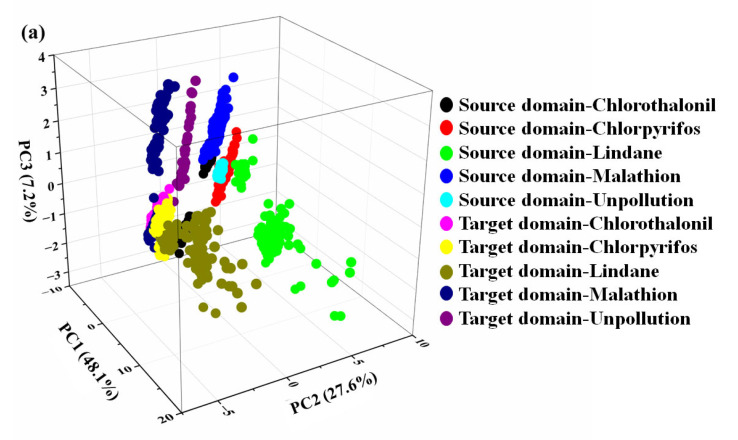

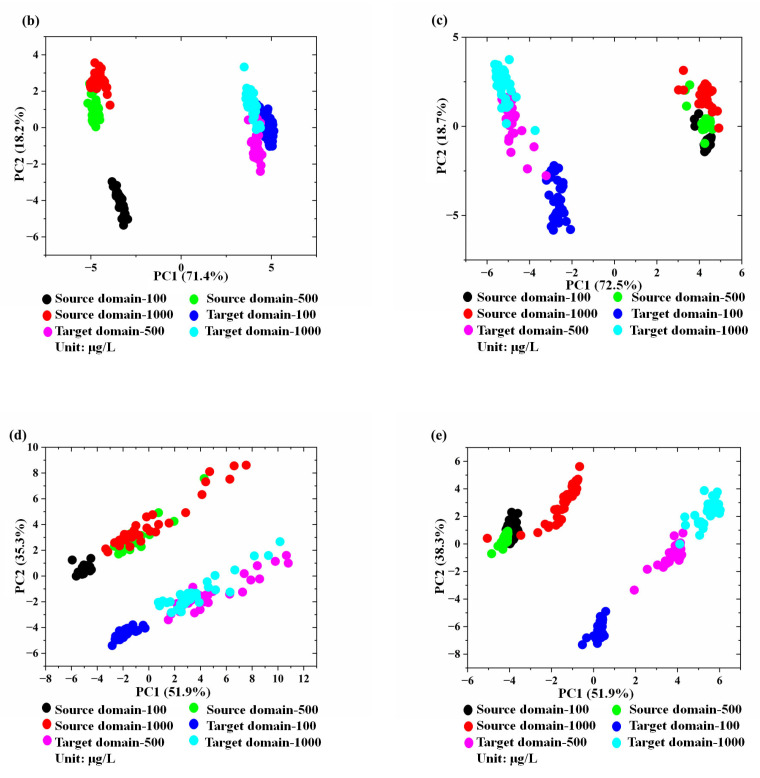
PCA plot performed on source domain and target domain samples. (**a**) qualitative analysis, (**b**) chlorothalonil semi-quantitative analysis, (**c**) chlorpyrifos semi-quantitative analysis, (**d**) lindane semi-quantitative analysis, (**e**) malathion semi-quantitative analysis.

Principal component analysis (PCA) is an unsupervised machine learning method [34,35]. In this study, PCA was used to visualize the differences between samples. The Mean feature extraction method was used to extract features from the original e-nose signals. These features were the input of PCA, and the output results of PCA are shown in Figure 5.

Figure 5 revealed that source and target domain samples were clearly separated into two clusters. Even in Figure 5a, a part of the source domain samples polluted by chlorothalonil partially overlapped with the target domain samples, but the overlapped regions were irrelevant to the samples polluted by chlorothalonil in the target domain samples. This outcome might be caused by various volatile compounds in the source and target domains (Table S2). Results from the PCA analysis showed that there were significant differences between the samples in the source and target domains. Thus, the prediction model constructed by sample data from the source domain to predict target domain samples might result in poor recognition accuracy. In the next work, we will try to introduce a transfer learning algorithm to solve this problem.

### 3.2. Selecting an Appropriate Feature Extraction Method

**Figure 6:**
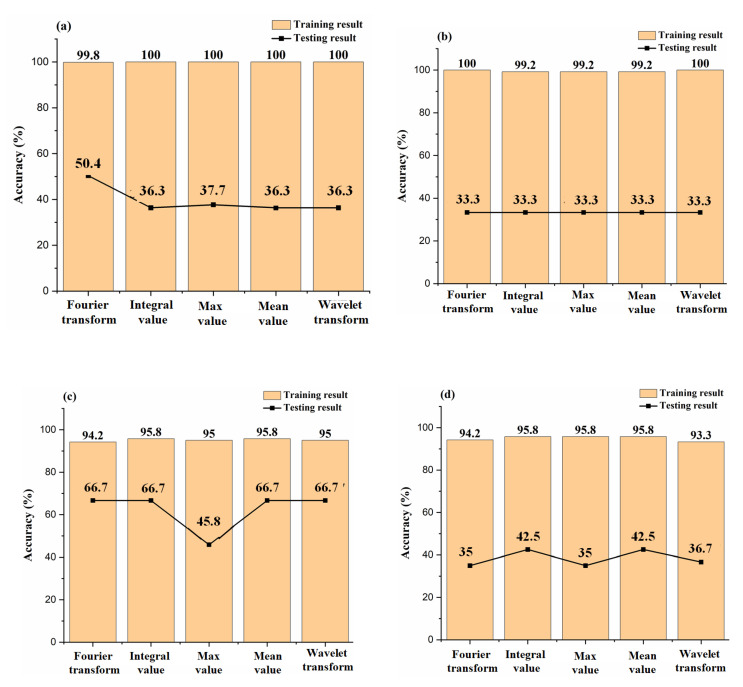

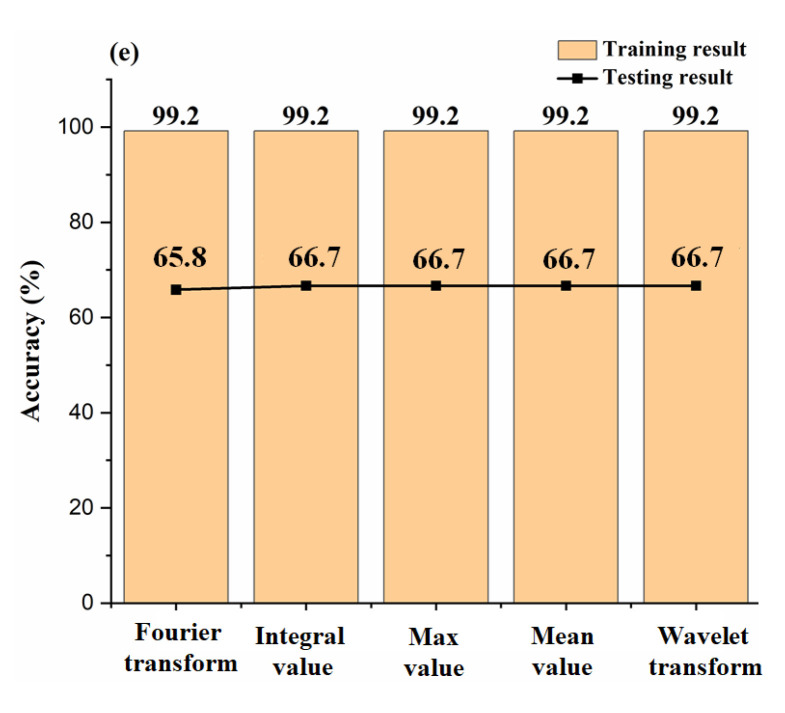
The recognition results of source domain sample models for target domain samples based on different feature extraction methods. (**a**) qualitative analysis, (**b**) chlorothalonil semi-quantitative analysis, (**c**) chlorpyrifos semi-quantitative analysis, (**d**) lindane semi-quantitative analysis, (**e**) malathion semi-quantitative analysis.

The selection of an appropriate feature extraction method plays an important role in improving the recognition accuracy of e-nose [36]. In this section, five feature extraction methods were used to extract features from the original signal of the e-nose. The five feature extraction methods were FT, IV, MAX, Mean, and WT. The features extracted from the source domain samples (Training set) were used to construct prediction models, and the machine learning algorithm used to build the prediction model was SVM. SVM was also used as the base classifier of the transfer learning algorithm later. The prediction model was used to predict the target domain samples (Testing set). The recognition accuracy of target domain samples was the key to selecting the appropriate feature extraction method. In this way, we conducted qualitative analysis and semi-quantitative analysis on pesticides in the samples, and the recognition results are shown in Figure 6.

As shown in Figure 6, the overall recognition accuracy of the training set samples was higher than 93.3%, and the prediction result accuracy of the testing set samples was lower than 66. 7%. From the experimental results, the prediction model established by the groundwater sample data from the source domain could not predict the pesticides in the target domain groundwater sample well; that was, the prediction model constructed by SVM did not have the ability to transfer. This result was consistent with the conclusion in Section 3.1. Although the prediction results of multiple prediction models on the target domain samples were poor, the recognition accuracy of each model was different. Of these, FT had the highest classification accuracy in qualitative analysis, while Mean and IV feature extraction methods had better effects in the semi-quantitative analysis. Compared with other feature extraction methods, FT, Mean, and IV feature extraction methods might be more suitable for data mining and processing. Thus, in the later work, the features extracted by the FT feature extraction method were used for qualitative transfer learning, and the features extracted by the Mean feature extraction method were used as the input of semi-quantitative transfer learning.

### 3.3. TrAdaBoost Transfer Learning Method for Qualitative Analysis

In order to evaluate the feasibility of the TrAdaBoost algorithm in improving the pesticide recognition accuracy in the target domain, this section mainly carried out two works: optimizing the parameters of the TrAdaBoost algorithm and comparing the recognition results of methods with transfer learning and without transfer learning. SVM was used as the base classifier for the TrAdaBoost algorithm.

#### 3.3.1. Optimizing the Parameters of the TrAdaBoost Method

**Figure 7:**
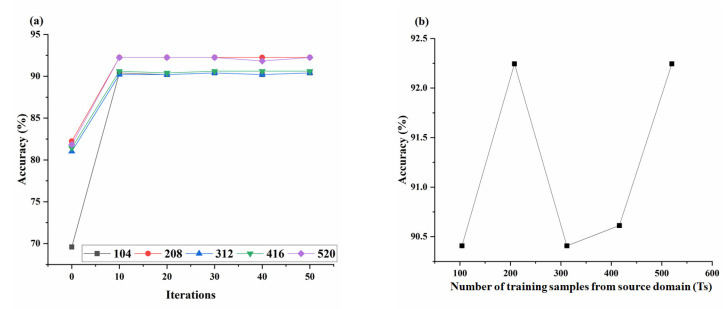
(**a**) The overall classification accuracies of TrAdaBoost based on SVM with different parameter settings of N and Ts. (**b**) shows the effect of the Ts on overall accuracy.

There are two parameters that need to be set in the TrAdaBoost algorithm, including the maximum iterations (N) and the number of training samples from the source domain (Ts) [25]. The different settings of both parameters will affect the recognition results. These two parameters were set to different values to improve the accuracy of the TrAdaBoost algorithm in recognizing pesticides in target domain samples. The experiments with different N values (i.e., 0, 10, 20, 30, 40, 50) and Ts values (i.e., 104, 208, 312, 416, 520) were tested. The number of training samples from the target domain (Tt) was fixed to 30, and the remaining 490 target domain samples (S) were used as a testing set. FT was the feature extraction method applied in this section. The overall performance of each combination of these two parameters is shown in Figure 7.

As shown in Figure 7a, the overall accuracy of recognition results obtained by the TrAdaBoost based on SVM showed a tendency to increase as the maximum number of iterations increases. It could be seen that the accuracy tends to be stable when the maximum iteration reaches 50. Based on Figure 7a, the accuracies of the TrAdaBoost based on SVM with the maximum iterations of 50 are illustrated in Figure 7b. With the increase of the Ts, the recognition accuracy curve achieved by the TrAdaBoost based on SVM showed a wavy growth trend. The changing trend of accuracy was related to whether the distribution of newly added samples and target domain samples was similar. When the new sample was similar to the target domain sample, the accuracy increased; otherwise, it would decrease. When Ts equaled 312 and 416, the low accuracies indicated a significant difference between the newly added samples and the target domain samples. The curve of recognition accuracy was similar to that in the literature [37]. When the Ts was set to 520, the recognition accuracy was the highest.

As mentioned above, when 520 of the training samples were from the source domain, and maximum iterations equaled 50, the performance of the TrAdaBoost was better than others. Thus, this combination was applied to the following experiments.

#### 3.3.2. Comparison of Different Methods

**Figure 8:**
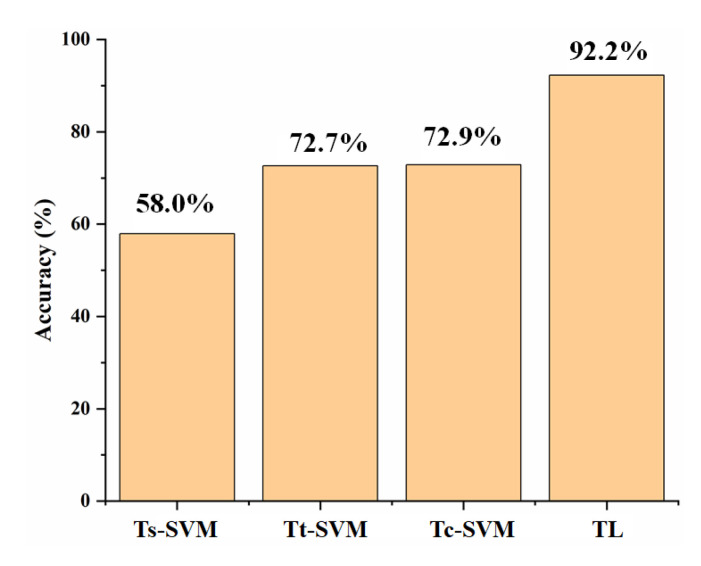
Accuracy comparison of the different methods. Ts-SVM and Tt-SVM represented SVM models trained by 520 samples from the source domain and 30 samples from the target domain, respectively; Tc-SVM represented the SVM model trained by the combination of Ts and Tt; TL represented the TrAdaBoost model based on SVM, and the training samples were consistent with Tc. The test samples for these four methods were the remaining target domain samples.

In this section, three groups of training samples (i.e., Ts, Tt, and Tc) were used to establish SVM models, respectively. Tc is the combination of two groups of samples (Ts and Tt). Different from transfer learning (TL), Ts was set to 520. The recognition accuracy of these models was compared with that of TL. The comparison result is shown in Figure 8.

As shown in Figure 8, the TrAdaBoost based on SVM was better than other methods without transfer learning when the samples from the target domain were limited. This was because the performances of the SVM algorithm depended mainly on the quantity and quality of Tt, which did not apply the prior knowledge of the Ts [38]. When the Ts were used for recognition solely, the recognition accuracy was the lowest because it did not contain enough useful information to predict the target domain samples. When the limited number of Tt was used solely for recognition, the classification accuracy was higher than that using the Ts but lower than that using the Tc. The reason might be that compared with Ts, Tt contained more sample information of the test set, so the recognition accuracy of the latter was higher than that of the former. Although the test set sample information contained in Ts was limited, it was still helpful for test set sample recognition. Thus, the classification accuracy of Tc was higher than that of Tt.

Compared with the methods without transfer learning, the transfer learning method obtained higher accuracies when using the same training samples. In the iterative process of TrAdaBoost, the weight of samples in the source domain that was similar to those in the target domain would increase and would decrease otherwise. Even for a small number of Tt, the results achieved by the TrAdaBoost approach based on SVM demonstrated an improvement in recognition accuracy, and the recognition accuracy was improved by 19.3% and reached 92.2%. These results highlighted the potential of the TrAdaBoost based on SVM for the recognition of pesticides in groundwater when the training samples were limited.

### 3.4. TrAdaBoost Transfer Learning Method for Semi-Quantitative Analysis

The TrAdaBoost approach based on SVM was applied to recognize pesticide concentration in groundwater. In this section, the workflow was consistent with the qualitative analysis. The difference was that the feature extraction method used in the semi-quantitative analysis was Mean, and the source and target domain samples were divided into four classes according to the pesticide type. Each type of pesticide in the source domain was used to assist in the identification of pesticide concentration in the corresponding target domain. Semi-quantitative analysis models of these four pesticides were used to construct and evaluate in this section.

#### 3.4.1. Optimizing the Parameters of the TrAdaBoost Method

**Figure 9:**
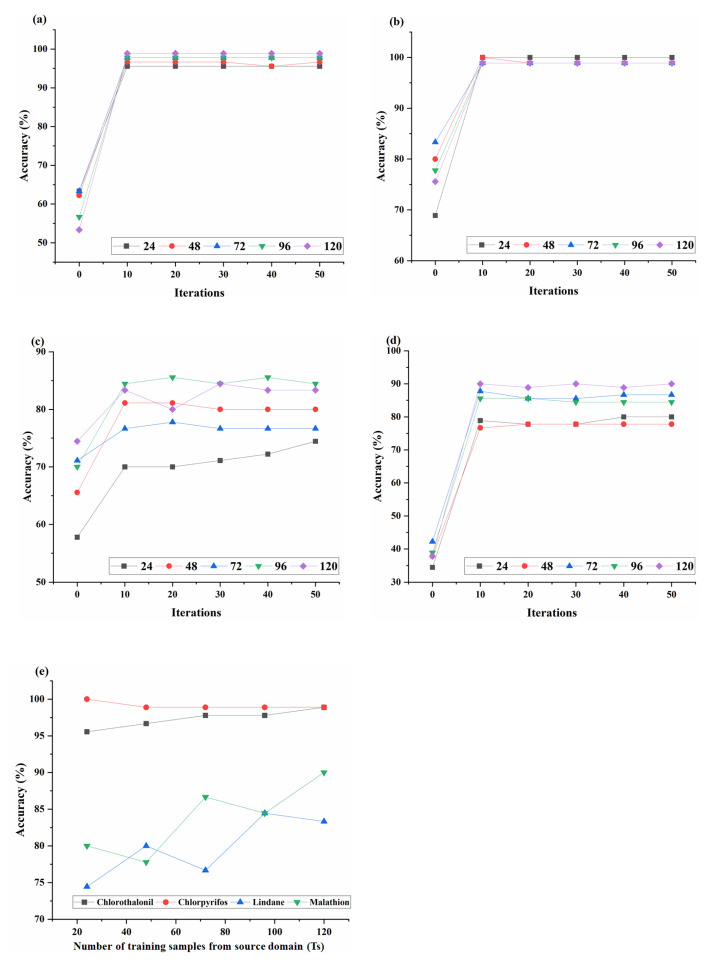
The overall classification accuracies of semi-quantitative analysis models with different parameter settings of N and Ts. (**a**) chlorothalonil, (**b**) chlorpyrifos, (**c**) lindane, (**d**) malathion. (**e**) shows the effect of the Ts on overall accuracy.

In the process of parameter optimization, N and Tt were consistent with those in Section 3.3.1. Since there were 120 samples of each class of pesticide in the source and target domain, Ts was set to 24, 48, 72, 96, and 120, and the number of S was 90. The construction and evaluation method of the semi-quantitative analysis model was consistent with that of the qualitative analysis model. The overall performance of each combination of these two parameters is shown in Figure 9.

As shown in Figure 9a–d, similar to the qualitative analysis, the recognition accuracy of the pesticide semi-quantitative analysis models also increased with the increase of the number of iterations and gradually became stable. When N was equal to 50, the relations between the recognition accuracy of TrAdaBoost for four pesticide concentrations and the increase of Ts were shown in Figure 9e. In Figure 9e, except chlorpyrifos, the concentration recognition accuracy of other pesticides also showed a wave-shaped growth trend with the increase of Ts. As for chlorpyrifos, the recognition accuracy was reduced to a certain extent. The reason might be that the newly added source domain samples could not provide more useful information for transfer learning, which on the contrary, introduced interference information.

As shown in Figure 9e, when Ts was set to 120, chlorothalonil and malathion had the highest recognition accuracy, while chlorpyrifos and lindane had the highest recognition accuracy when Ts was set to 24 and 96, respectively. Thus, these combination recognition accuracies were applied to compare with the results of the method without transfer learning.

#### 3.4.2. Comparison of Different Methods

**Figure 10:**
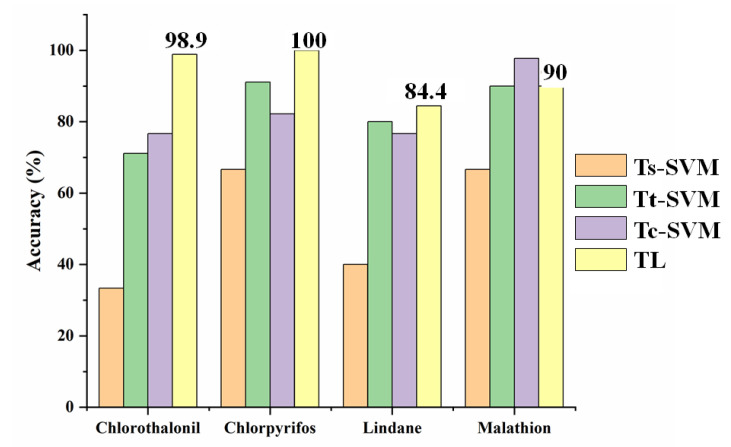
Accuracy comparison of the different methods. Ts-SVM and Tt-SVM represented SVM models trained by 120 samples from the source domain and 30 samples from the target domain, respectively; Tc-SVM represented the SVM model trained by the combination of Ts and Tt; TL represented the TrAdaBoost model based on SVM. The test samples for these methods were the remaining target domain samples.

These four pesticides were applied for semi-quantitative analysis by the method without transfer learning. Three groups of training samples (Ts, Tt, and Tc) were used as the input of SVM. In the method without transfer learning, Ts, Tt, and Tc were set to 120, 30, and 150, respectively. The recognition accuracies of methods with transfer learning and without transfer learning are listed in Figure 10.

As shown in Figure 10, the same as qualitative analysis, when Ts was used for classification solely, the classification accuracy was the lowest. The differences were that (1) the classification accuracy of the Tc might be lower than that of Tt, and (2) the classification accuracy of the TL was lower than that of Tc. As mentioned in Section 3.4.1, when the newly added source domain samples could not provide useful information, the increase of source domain samples would reduce the recognition accuracy; thus, the recognition accuracy of Tc might be lower than that of Tt (lindane and chlorpyrifos). In the semi-quantitative analysis model of malathion, the recognition accuracy of TL was lower than that of Tc. It was possible that when the training data set was sufficient to construct a model with ideal generalization performance, the introduction of transfer learning might reduce the recognition accuracy [39].

As mentioned above, although for the semi-quantitative analysis of malathion, the recognition accuracy of the TrAdaBoost method was lower than that of the method without transfer learning, the recognition accuracy of this method itself reached 90%. In addition, for the semi-quantitative analysis of the other three pesticides, the recognition accuracy of the TrAdaBoost method was higher than that of the method without transfer learning. Especially in the semi-quantitative analysis of chlorothalonil, the recognition accuracy could be increased by 22.2%. Thus, the TrAdaBoost method based on SVM is suitable for the recognition of pesticide concentration in groundwater when the training samples are limited.

## 4. Conclusions

This paper proposed a method (TrAdaBoost) to improve the recognition accuracy of e-nose when the training samples are limited. As far as we know, this is also the first report to try to solve the difficulty of sample recognition due to different domains. By comparing with the method without transfer learning, the performance of this method was superior. In the process of qualitative and semi-quantitative analysis, the recognition accuracy could be improved by 19.3% and 22.2%, respectively. The proposed method could reduce the dependence on the number of target samples and save the sampling time and cost on the basis of making full use of the past sample information. This has an important impact on accelerating the application of e-noses in the detection of pesticides in groundwater. In addition, this method may also provide some reference value for other e-nose applications that face the problem of recognition difficulty due to limited samples.

There are also some uncertainties and limitations to the TrAdaBoost method. (1) This method needs to find a suitable source domain. Thus, before using this method, it is necessary to determine whether the response signals of the groundwater samples in the area to be tested are similar to those of the source domain samples; (2) this study used the simulated groundwater samples in the experiment. Thus, the recognition accuracy of this method for real groundwater samples cannot be determined. In future work, it is necessary to explore new transfer learning methods for groundwater pesticide prediction. Collecting real groundwater samples to test the recognition ability of the proposed method is also needed to be conducted.

## Figures and Tables

**Table 1:** Properties of soil samples.

Soil Sample	Organic Matter (g/kg)	PH	Cation Exchange Capacity (cmol+/kg)
location 1	10.1	6.7	7.4
location 2	47.4	7.2	14.6

## Data Availability

Not applicable.

## Supplementary Materials

The following supporting information can be downloaded at: https://www.mdpi.com/article/10.3390/s23083856/s1, Table S1: the volatile compounds in pesticide samples; Table S2: the main volatile compounds in simulated groundwater samples 1 and 2; Table S3: Properties of gas sensors chosen in the e-nose system.
