# Supplementary material for: Improving Recognition Accuracy of Pesticides in Groundwater by Applying TrAdaBoost Transfer Learning Method
Source: Sensors (Basel). 2023 Apr 10;23(8):3856. doi: 10.3390/s23083856 (PMC10143876; doi:10.3390/s23083856)
Supplement: Supplementary file 1 [file sensors-23-03856-s001.zip › sensors-2284703-supplementary.pdf]

Supporting Information for:

Improving Recognition Accuracy of Pesticides in Groundwater by Applying TrAdaBoost Transfer Learning Method

Donghui Chen [1,2,3], Bingyang Wang [1,2,3], Xiao Yang [1,2,3], Xiaohui Weng [3,4] and Zhiyong Chang [1,2,3,*]

[1] Key Laboratory of Bionic Engineering, Ministry of Education, Jilin University, Changchun 130022, China
[2] College of Biological and Agricultural Engineering, Jilin University, Changchun 130022, China
[3] Weihai Institute for Bionics, Jilin University, Weihai 264401, China
[4] School of Mechanical and Aerospace Engineering, Jilin University, Changchun 130022, China
* Correspondence: zychang@jlu.edu.cn

Table S1. The volatile compounds in pesticide samples.

chlorothalonil		chlorpyrifos		malathion		lindane
Decane	Tetradecane	Ethylbenzene	Chlorpyrifos	Ethylbenzol	Azulene	Toluene
Tridecane	Ditan	Mellitene	Chlorobenzene	Isocumene	m-Ethylcumene	Lindane
Dodecane	Dihydrocurcumene	o-Ethyltoluene	p-Ethylcumene	p-Ethyltoluene	4-Ethyl-m-xylene	
Naphthalene	Mellitene	Isobutylbenzene	3-o-Tolyl-1-butene	2-Ethyltoluene	2,5-Diethyltoluene	
Hexylcyclohexane	2-Isopropylnaphthalene	2-Phenylbutane	4,4'-Dimethylbiphenyl	p-Cymene	p-Ethylcumene	
2,4,5-Trimethylcumene	2,6-Diisopropylnaphthalene	Hemimellitol	2-Methylnaphthalene	Indane	4,6-Dimethylindan	
2-Methyldodecane	1,3,5-Triisopropylbenzene	p-Cymene	2,5-Diethyltoluene	3-Propyltoluene	2-Methylnaphthalene	
4,7-Dimethylindan	trans-Verbenyl isovalerate	Indane	3,4-Dimethylcumene	o-Diethylbenzene	Trimethyl thiono-phosphate	
7-Methyltridecane	Pyrocatechitol	2-Propyltoluene		4-Ethyl-o-xylene	Methyl phosphoro-dithioate	
Muurolane	(Z)-7-Hexadecenal	1-Methylindan		2-Propyltoluene		
1-Tridecene	4,7-Dimethylbenzofuran	2-Allyltoluene		o-Cymene		
3-Methylundecane	Landrin	3,5-Diethyltoluene		2-Phenylpentane		
1,3-Di-iso-propyl-5-ethylbenzene	Phthalodinitrile	(2-Methylcyclopropyl)-benzene		(2-Methylcyclopropyl)-benzene		
Dicyclohexyl	Cetyl chloride	4-Ethyl-m-xylene		2-Allyltoluene		
Heptylcyclohexane	Chlorothalonil	p-Ethyltoluene		3,5-Diethyltoluene		
2-Methyltridecane	2,4-Di-tert-butylphenol	2-Ethylnaphthalene		2-p-Tolylbutane		
3-Methyltridecane		Azulene		2,4-Diethyltoluene		

Table S2. The main volatile compounds in simulated groundwater samples 1 and 2.

Simulated groundwater sample 1			Simulated groundwater sample 2
Toluene	Benzophenone	Butyl ether	Nonanal
Nonanal	Texanol isobutyrate	2-(4-Phenoxyphenyl)indolizine	Decanal
Decanal	Isobutyraldehyde trimer		Dibutyl phthalate
2-Ethylhexanol	1,2-Cyclohexanediol		Diisobutyl phthalate
Dibutyl phthalate	Butyl Oxitol glycol ether		1,2-Cyclohexanediol

Table S3. Properties of gas sensors chosen in the e-nose system.

Serial number	sensor model	sensor sensitivity	Sensitivity
S1	TGS2600	alcohol, H_2	1-30ppm
S2	TGS2602	VOCs, hydrogen sulfide, ammonia	1-30ppm
S3	TGS2603	air pollutant	1-10ppm
S4	TGS2610	LP gas	500-10000ppm
S5	TGS2611	CH_4	500-10000ppm
S6	TGS2612	CH_4, C_3H_8, C_4H_{10}	400-1000ppm
S7	TGS2618	C_4H_{10}, LP gas	500-10000ppm
S8	TGS2620	ethanol, organic solvent	50-5000ppm
S9	GSBT11	toluene, formaldehyde, benzene	0.1-10ppm
S10	MS1100	VOCs, organic compounds, smoke	5-1000ppm
S11	MP-2	C_3H_8, smoke	200-10000ppm
S12	MP-4	CH_4, natural gas, biogas	300-10000ppm
S13	MP-5	liquefied petroleum gas	300-10000ppm
S14	MP-7	CO	50-1000ppm
S15	MP-9	CO, CH_4	50-1000ppm
S16	MP-3B	alcohol	0-500ppm
S17	MP135	H_2, alcohol, CO	10-500ppm
S18	MP402	CH_4, natural gas, biogas	300-10000ppm
S19	MP503	alcohol, smoke, C_4H_{10}, formaldehyde	1-1000ppm
S20	MP702	NH_3	0-100ppm
S21	MP801	benzene, toluene, formaldehyde, alcohol, smoke	0.5-1000ppm
S22	MP901	benzene, acetone, lighter gas, paint	10-1000ppm
S23	MP905	benzene, toluene, formaldehyde, lighter gas	0.5-1000ppm
S24	WSP1110	NO_2	0.1-10ppm
S25	WSP2110	toluene, formaldehyde, benzene, alcohol, acetone	1-50ppm
S26	WSP7110	hydrogen sulfide, acetylene, benzene	0-50ppm
